# Breast cancer survival and the expression of genes related to alcohol drinking

**DOI:** 10.1371/journal.pone.0228957

**Published:** 2020-02-20

**Authors:** Hui G. Cheng, Agustin Gonzalez-Reymundez, Irene Li, Ania Pathak, Dorothy R. Pathak, Gustavo de los Campos, Ana Ines Vazquez

**Affiliations:** 1 Department of Epidemiology & Biostatistics, Michigan State University, MI, United States of America; 2 The Institute for Quantitative Health Science and Engineering, Michigan State University, MI, United States of America; Florida State University, UNITED STATES

## Abstract

Breast cancer is the leading cause of cancer-related disease in women. Cumulative evidence supports a causal role of alcohol intake and breast cancer incidence. In this study, we explore the change on expression of genes involved in the biological pathways through which alcohol has been hypothesized to impact breast cancer risk, to shed new insights on possible mechanisms affecting the survival of breast cancer patients. Here, we performed differential expression analysis at individual genes and gene set levels, respectively, across survival and breast cancer subtype data. Information about postdiagnosis breast cancer survival was obtained from 1977 Caucasian female participants in the Molecular Taxonomy of Breast Cancer International Consortium. Expression of 16 genes that have been linked in the literature to the hypothesized alcohol-breast cancer pathways, were examined. We found that the expression of 9 out of 16 genes under study were associated with cancer survival within the first 4 years of diagnosis. Results from gene set analysis confirmed a significant differential expression of these genes as a whole too. Although alcohol consumption is not analyzed, nor available for this dataset, we believe that further study on these genes could provide important information for clinical recommendations about potential impact of alcohol drinking on breast cancer survival.

## Introduction

Breast cancer is the leading cause of cancer-related disease burden in women with an estimated 464,000 deaths worldwide [1]. Cumulative evidence supports a causal role of alcohol intake and breast cancer incidence [2–4]. Potential mechanisms include both global and site-specific changes induced by alcohol intake [5]. Nonetheless, evidence about alcohol consumption and breast cancer survival, an inherent element in cancer-related disease burden, has been mixed. Therefore, whether the observed pattern is a reflection of true causal relationship is unknow. For example, it is well possible that the observed relationships are confounded by self selection biases (e.g., “healthy drinker effect”) [6]. Given this context, it is of interest to examine the relationship between genes related to alcohol consumption and breast cancer survival, in order to shed new light on potential underlying mechanisms linking alcohol consumption prior to diagnosis and breast cancer survival.

Against this background, we seek to estimate the relationships linking the expression of genes related to alcohol drinking and breast cancer survival using a large database with modern quantitative genomics methods [7]. Our literature review identified the following groups of alcohol-related genes: genes involved in alcohol metabolism (*ALDH2*, *ADH1C*, *CYP2E1*, *AOX1*), DNA repair (*XDH*, *XRCC1*, *ERCC2*, *XPC*, *OGG1*), reactive oxygen species metabolism (*CBS*, *SOD2*, *GSTP1*), estrogen metabolism (*CYP1B1*, *COMT*), cell-to-cell contact (*ITGA5*) and folate metabolism (*MTHFR*) [8–11]. We first estimate the associations between the expression of each individual gene with breast cancer survival. Because sets of genes are often up- or down-regulated together, we conducted a gene set enrichment analysis (GSEA) to further elucidate whether the above mentioned genes were significanctly up or downregulated in women with shorter or longer survival.

## Materials and methods

### Study population and sample

METABRIC: Patient data consisted of a sample of the Molecular Taxonomy of Breast Cancer International Consortium (METABRIC) data set [12,13] a 4-year retrospective cohort study with survival information from 1,977 breast cancer cases (1,415 luminal, 431 triple negatives, and 131 basal subtypes) of white Caucasian women between 21 and 96 years of age (median, 62 years of age). From this sample, a group of 1,674 patients received threatment in the form of radiotherapy, chemotherapy, and/or hormonotherapy. Samples correspond to biopsies from primary tumor (breast) conserved as frozen tissue, and taken before patients underwent treatment. A total of 284 deaths occurred within four years after diagnosis (based on the time of maximum prediction accuracy of survival time for this data set [14], across different cancer subtypes: 131 luminal (40% deceased at fourth year), 431 triple negative (28%) and 131 Her2+ subtype (10%)). In this study, tumor grade was grade one (n = 950), grade two (n = 775), and grade three (n = 169), while tumor sizes ranged from 0.17 to 1.82 cm of diameter. Gene expression profiles from the tumor biopsies were normalized by the intensity of 49,473 microarray Illumina HT-12 v3 probes [12] and summarized at the gene level by the WGCNA R package [15]. Genes with more than 20% of missing data or zero variability were removed. The remaining missing data were replaced by the mean. The final number of genes after quality controls was 19,535. More detail about cohort and edition criteria can be found elsewhere [12,14]. The current study uses anonymized data accessed through *Synapse* (https://www.synapse.org/Portal.html#!Synapse:syn1710250/wiki/27348). Study was determined not “human subjects” by the Institutional Review Board (IRB) of Michigan State University (Study ID: STUDY00000996). Funding for METABRIC was provided by Cancer Research UK and the British Columbia Cancer Agency Branch. Normalized gene expression, clinical and survival data can be accessed from European Genome-phenome Archive (EGA, study accession EGAS00000000083) upon approval by METABRIC data access committee TCGA: The Cancer Genome Atlas ([16], n = 506) was used for validation. The dataset is younger than METABRIC and only a third of it has already 4 years of followup. In this dataset 437 patients were alive at 4 years. Covariables consisted of: age at diagnosis between 27 and 90 years old (median = 59 years), cancer subtype (0.18% triple negative, 78% luminal, 0.08% Her2+). Only 40% of patients in this dataset received some form of treatment. Gene expression count for the 18,322 genes was estimated from log RPKM counts from RNA-Seq obtained from Illumina HiSeq RNA V2 platform [17].

### Statistical analysis

First, we performed a step-forward variable selection on a model including the median expression across all genes as response, using vital status, treatment, cancer subtype, tumor size, tumor grade and age as covariates. Next, we estimated the relationship between the expression of each gene in the data set and the selected covariates using the following statistical model followed by ANOVA:

$${y^{\prime }}_{g}=\mu_{g}+\alpha_{g}S+\beta_{g}C+\gamma_{g}A+\delta_{g}SxC+\zeta_{g}AxC+\eta_{g}SxAxC+\varepsilon_{g}$$ Eq [1]

where ***y***_*g*_ is the expression of the gene *g* (i.e. the expression level of the gene *g* across samples), *μ_g_* is the general mean for the gene *g*, and ***S, A, C, A*x*C, S*x*C*** and ***S*x*A*x*C*** are survival status (***S***), age (***A***) cancer subtype (***C***), and the interactions between all terms (the Greek letters are the corresponding effects) (see results). The expression of gene *g* was considered associated with the survival of breast cancer when the estimated *α_g_* was statistically significant based on p-values adjusted for multiple testing using the False Discovery Rate (FDR) method across all genes [18].

Next, to estimate the potential effect of groups of genes as aggregates, we performed a Gene Set Enrichment Analysis (GSEA) with the Limma R package [19]. The method starts by ranking the target genes based on their ${\hat{\alpha }}_{g}$ amongst the effects of the remaining genes. Then, a score is estimated, where the maximum can be considered as a modified version of the Kolmogorov-Smirnov (KS) statistics, weighted by the ${\hat{\alpha }}_{g}$ of each gene. The score obtained gives evidence to determine if the distribution of target genes differs or resembles the distribution of the remaining genes in the genome. A p-value was then obtained from a permutation test based on 10,000 iterations. Finally, if the target genes cluster on either the top or bottom of the list, the target genes would be considered as up- or down-regulated as a group.

### Validation

Model described in Eq [1] was also adjusted in TCGA data. The p-values for survival are reported as validation of results.

## Results

**Fig 1:**
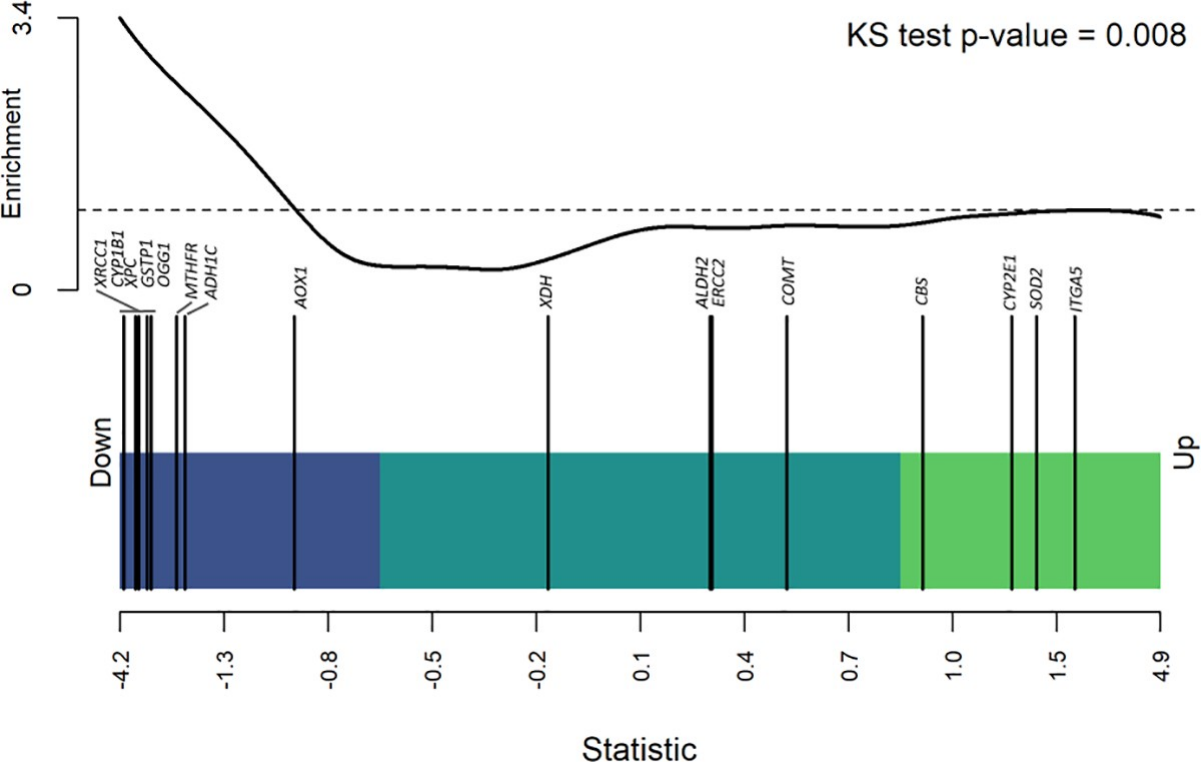
Gene-set enrichment analysis for gene expression across vital status. The *x*-axis corresponds with the sorted effects of vital status, estimated assuming the following probabilistic model ${y^{\prime }}_{g}=\mu_{g}+\alpha_{g}S+\beta_{g}C+\gamma_{g}A+\delta_{g}SxC+\zeta_{g}AxC+\eta_{g}SxAxC+\varepsilon_{g}$ for the expression of 19,535 genes. The light green and blue rectangles represent the up and down regulation regions defined by the third and first quartile, respectively. The vertical bars represent a target set of genes involved in alcohol metabolism. The black curve shows the estimated running enrichment score, and the dashed line shows the threshold for random disposition of the target set. The p-value was obtained by modified Kolmogorov-Smirnov (KS) test over the maximum value of the enrichment score. Genes in the target set: *XRCC1*, *CYP1B1*, *XPC*, *GSTP1*, *OGG1*, *MTHFR*, *ADH1C*, *AOX1*, *XDH*, *ALDH2*, *ERCC2*, *COMT*, *CBS*, *CYP2E1*, *SOD2*, and *ITGA5*.

The significant covariates resulting from the variable selection procedure were vital status (**S**), cancer subtype (**C**), and age (**A**). These covariates and all their interactions were then tested with ANOVA in the models that have the expression of each gene as response. As shown in Table 1, nine genes had differencial expression between alive and death women with breast cancer from the 16 genes in the alcohol pathway. Three genes were up-regulated (i.e., *ITGA5*, *CBS*, and *SOD2*), while six were down-regulated (*i*.*e*, *XDH*, *XRCC1*, *MTHFR*, *CYP1B1*, *XPC*, and *GSTP1*) among individuals who died of breast cancer. Robust differences in the expressions of most of the genes under study were observed across the three cancer subtypes. Fig 1 shows the results of the GSEA, in terms of the distribution of our target genes involved in alcohol consumption and breast cancer survival, against the expression level of the remaining genes in our data set. The plot shows that our target set of genes was significantly downregulated as a whole (p-value = 0.008), with most of its genes clustered at the bottom of the sorted vital status’ effects (${\hat{\alpha }}_{g}$). No statistically significant interactions of gene expression were found between survival, age and cancer subtypes.

**Table 1:** ANOVA results for the expression of genes related to alcohol metabolism. HUGO gene names and functions are presented along with gene expression effect on survival (${\hat{\alpha }}_{g}$). The effect shows if the gene was up- or down-regulated in tumors from deceased patients at the fourth year. The p-values for a two-way ANOVA (FDR-adjusted across all genes) are also shown. ANOVA tests considered the effect of gene expression on vital status (S), cancer subtype (C), and patients age (A). Same models were validated on breast cancer data from The Cancer Genome Atlas (TCGA).

				Adjusted FDR p-values			
		${\hat{\alpha }}_{g}$		METABRIC			TCGA
Genes	Function	METABRIC	TCGA	S	C	A	S
***XRCC1***	DNA repair	-2.87	1.78	<1e-3	<1e-3	0.42	--
***CYP1B1***	Estrogen Metabolism	-2.31	1.19	<1e-3	<1e-3	<1e-3	--
***XPC***	DNA repair	-2.20	-0.16	<1e-3	<1e-3	0.01	0.03
***GSTP1***	ROS	-2.02	-0.18	<1e-3	<1e-3	<1e-3	0.01
***OGG1***	DNA repair	-1.95	-0.01	0.41	0.00	0.37	--
***MTHFR***	Folate Metabolism	-1.65	1.07	<1e-3	<1e-3	0.21	--
***ADH1C***	Alcohol Metabolism	-1.57	1.41	0.69	0.06	<1e-3	--
***AOX1***	Alcohol Metabolism	-0.93	-0.69	0.10	0.00	0.67	--
***XDH***	DNA repair	-0.13	-0.13	<1e-3	<1e-3	0.45	--
***ALDH2***	Alcohol Metabolism	0.29	-0.00	0.27	<1e-3	0.99	--
***ERCC2***	DNA repair	0.30	0.37	0.08	0.14	0.83	--
***COMT***	Estrogen Metabolism	0.50	0.07	0.73	<1e-3	0.51	--
***CBS***	ROS	0.93	0.26	<1e-3	<1e-3	0.48	--
***CYP2E1***	Estrogen Metabolism	1.28	-1.52	0.09	<1e-3	0.17	--
***SOD2***	ROS	1.41	-0.71	<1e-3	<1e-3	0.43	--
***ITGA5***	Cell-to-cell contact	1.64	0.37	<1e-3	<1e-3	0.27	0.05

**Fig 2:**
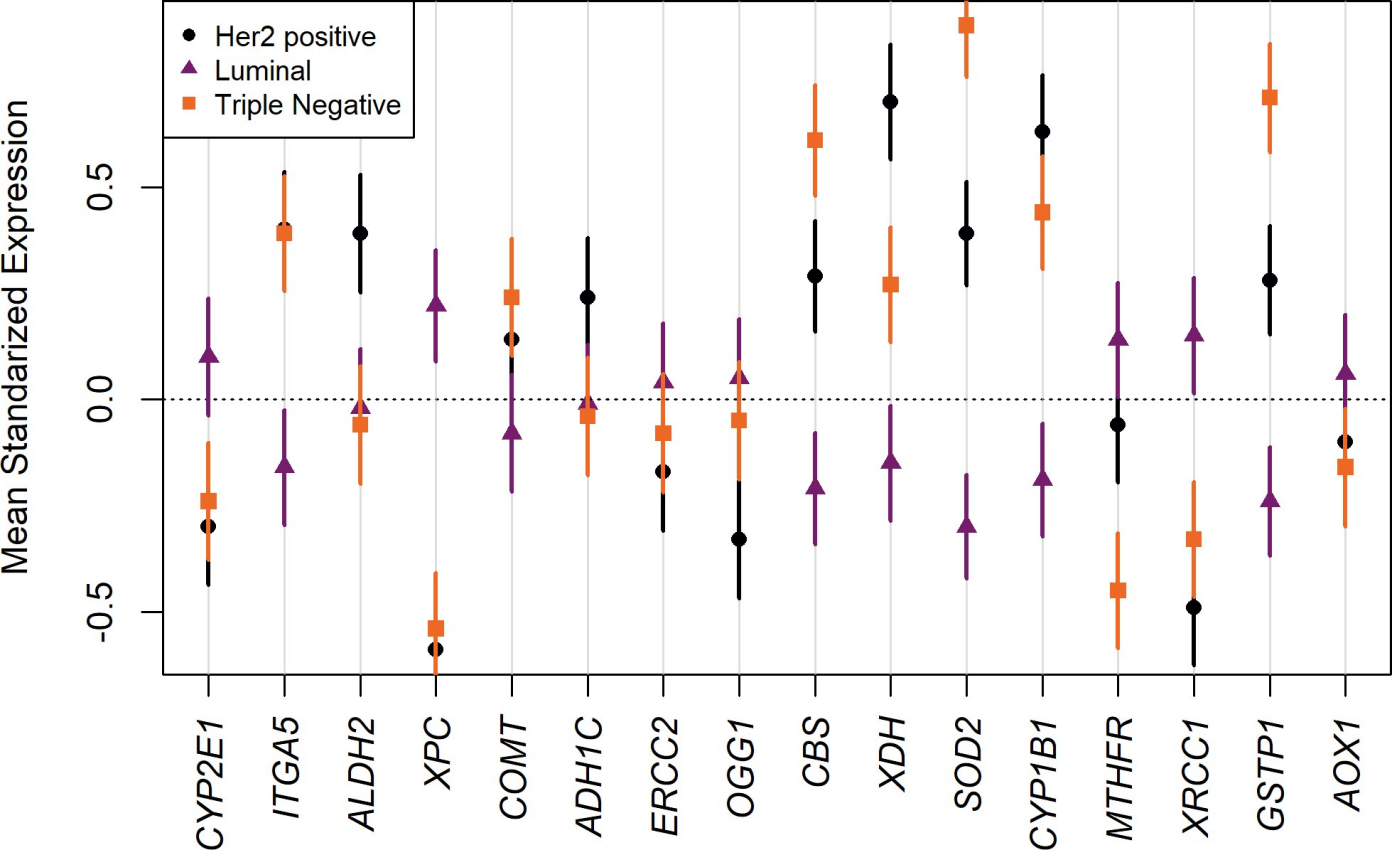
Target genes mean expression by cancer subtype. Bonferroni intervals were obtained considering the total number of pair-wise comparisons among all genes in the datasets. Intervals for alcohol related genes are plotted around the mean expression by cancer subtype.

Fig 2 shows a visual representation of gene expressions per gene and by cancer subtypes. Cancer subtype based on our ANOVA results (Table 1) was significantly associated with the expression of all but 2 (*ADH1C* and *ERCC2*) alcohol-breast cancer risk related genes. The figure shows the direction of the expression for each subtype. The figure also suggests that the triple negative (TN) subtype was the group with largest inter-individual variation in gene expression, as compared to the other subtypes.

From the 9 genes significant in the Metabric dataset, genes *XPC*, *GSTP1*, and *ITGA5* were validated as statistically significant genes for survival in TCGA, and exhibiting the same direction of effects than in the Metabric dataset (Table 1). However, not all the significant genes in Metabric exhibited the same direction of effects in TCGA. The lack of validation of the other 6 genes, as well as incongruences in some of the effects directions, may indicate that either they are in fact not related to survival, or it could be simply lack of power due to the lower sample size of TCGA (n = 506) as compared to METABRIC (n = 1,977).

## Discussion

In this study, we found that the expression of 9 out of 16 alcohol-related genes at the primary tumor were associated with breast cancer four-year survival, after correcting by cancer subtype and age, and 3 of these genes actually validate in a much smaller dataset. Four-year survival is a time point where maximum prediction accuracy was obtained with gene expression from breast cancer patients [14]. No evidence was found of interaction between the expression in different cancer-subtype, age and vital status.

It is known that alcohol consumption can increase the concentration of ROS and acetaldehyde, factors that can damage DNA and generate cancer-inducing mutations. Downregulation of *GSTP1* (gene involved in ROS metabolism) can elevate the concentration of ROS. This, together with dowregulation of genes involved in DNA repair (e.g., *XDH*, and *XRCC1*) might enhance the noxious effects of oxidative agents. Decreased expression of *CBS* and *MTHFR* has been associated with increased levels of homocysteine in breast, which might also increase the risk of cancer [20]. However, CBS has been also reported as having oncogenic properties in breast[21], as well as in colon and ovarian cancers[22]. Higher levels of *CYP2E1* have been linked with the production of procarcinogenics [23], while upregulation of *ITGA5* is known to be involved in higher invasiveness of tumor cells [24]. From these group of genes, *XPC*, *GSTP1*, and *ITGA5* were validated on breats cancer samples from TCGA. These genes may provide potential target for future studies on the mechanisms linking alcohol consumption, breast cancer risk and subsequent survival post diagnosis

The downregulation of *CYP1B1* in patients of shorter survival was unexpected. This gene is involved in the activation of pro-carcinogens, so we would expect the opposite direction in their expression. Here, 14 out of the 16 genes were differentially expressed across cancer subtypes. Differences were mostly between triple-negative and other subtypes. Due to the non-significant interactions between gene expression across vital statuses and cancer subtype (Table 1), this pattern migh be explained by the different molecular heterogeneity exhibited by each subtype, with triple negatives being notoriously heterogenous [25].

Implications of this study should be interpreted with the following limitations. This study does not include alcohol consumption level, thus gene expression level of alcohol-related genes may be up or down regulated due to either alcohol consumption or other factors. Finally, alcohol can induce a wide range of reactions after ingestion. Our list of genes is not exhaustive of all genes involved in these reactions. Future studies with an unbiased approach may provide more complete pictures for the alcohol-breast cancer risk -survival relationship.

In this study, we provide strong evidence linking the expression of 9 genes that are listed as involved in alcohol metabolism and breast cancer survival (*XRCC1*, *CYP1B1*, *XPC*, *GSTP1*, *MTHFR*, *XDH*, *CBS*, *SOD2*, and *ITGA5*) with FDR adjusted p-values lower than 1e-3. Three of these genes are validated in an independent dataset. Future studies associating alcohol consumption before and after breast cancer diagnosis will be able to connect alcohol consumption and level of consumption with gene expression values in breast tissue, providing information for clinical recommendations about alcohol consumption among breast cancers survivors.”
